# Polaritonic Chemistry Using the Density Matrix Renormalization Group Method

**DOI:** 10.1021/acs.jctc.4c00986

**Published:** 2024-10-23

**Authors:** Mikuláš Matoušek, Nam Vu, Niranjan Govind, Jonathan J. Foley, Libor Veis

**Affiliations:** † J. Heyrovský Institute of Physical Chemistry, Academy of Sciences of the Czech Republic, v.v.i., Dolejškova 3, 18223 Prague 8, Czech Republic; ‡ Faculty of Mathematics and Physics, Charles University, 12116 Prague 2, Czech Republic; ¶ Department of Chemistry, University of North Carolina Charlotte, Charlotte, North Carolina 28223, United States; § Physical and Computational Sciences Directorate, Pacific Northwest National Laboratory, Richland, Washington 99352, United States; ∥ Department of Chemistry, University of Washington, Seattle, Washington 98195, United States

## Abstract

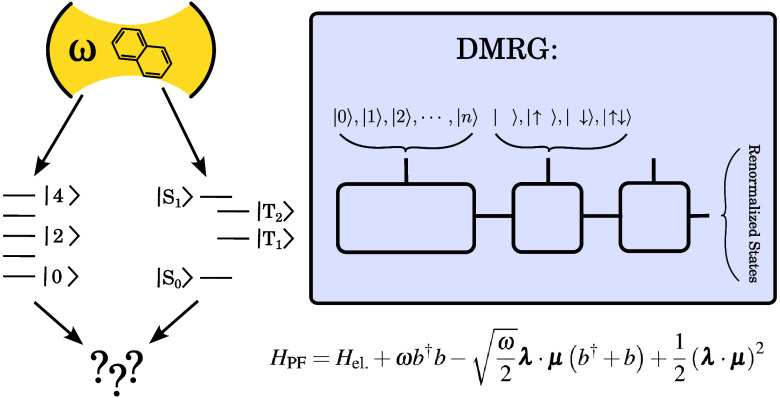

The emerging field of polaritonic chemistry explores the behavior of molecules under strong coupling with cavity modes. Despite recent developments in *ab initio* polaritonic methods for simulating polaritonic chemistry under electronic strong coupling, their capabilities are limited, especially in cases where the molecule also features strong electronic correlation. To bridge this gap, we have developed a novel method for cavity QED calculations utilizing the Density Matrix Renormalization Group (DMRG) algorithm in conjunction with the Pauli–Fierz Hamiltonian. Our approach is applied to investigate the effect of the cavity on the S_0_–S_1_ transition of *n*-oligoacenes, with *n* ranging from 2 to 5, encompassing 22 fully correlated π orbitals in the largest pentacene molecule. Our findings indicate that the influence of the cavity intensifies with larger acenes. Additionally, we demonstrate that, unlike the full determinantal representation, DMRG efficiently optimizes and eliminates excess photonic degrees of freedom, resulting in an asymptotically constant computational cost as the photonic basis increases.

## 1 Introduction

Photonic cavities are an excellent tool for studying the interactions between the quantized electromagnetic field and matter. Originally starting with Rydberg atoms,^1,2^ experimental developments enabled the field to move to larger systems, such as organic chromophores.^3^ This opened a myriad of new potential applications, including polaritonic chemistry, where chemical reactions are modified through strong coupling between cavity modes and molecular electronic or vibrational degrees of freedom.^4−7^ These developments go hand in hand with the need for accurate theoretical methods, allowing us to be confident in both our predictive abilities and the interpretation of experimental results.^8^

A detailed understanding of molecular structure and dynamics when cavity modes strongly couple to molecular electronic degrees of freedom (i.e., electronic strong coupling) requires that both electronic and photonic degrees of freedom are treated on equal quantum mechanical footing. One route that has been pursued by several groups recently includes generalizing the tools of *ab initio* electronic structure theory to explicitly include coupling to quantized photonic degrees of freedom. Such approaches have included quantum electrodynamics generalizations of density functional theory (QEDFT^9−15^ and QED-DFT^16−18^), real-time^9,10,19−22^ and linear-response^16,23,24^ formulations of QED-TDDFT, configuration interaction (QED-CIS),^25,26^ cavity QED extension of second-order Møller–Plesset perturbation theory and the algebraic diagrammatic construction,^27,28^ coupled cluster (QED-CC),^18,29−31^ variational QED-2-RDM methods,^32^ and diffusion quantum Monte Carlo (QMC).^33^ Many of the aforementioned methods, however, cannot properly describe molecules with a multireference character caused by strong electronic correlation. Unfortunately, many important chemical problems fall into this category, e.g. homolytic bond breaking/formation, open-shell and excited electronic states, transition-metal complexes, and transition states of chemical reactions. The last mentioned category is especially striking, since the idea behind polaritonic chemistry is to use an optical cavity coupling to alter chemical reactions, potentially by modifying the transition states or transition pathways, or by modifying or introducing intersections or near degeneracies between electronic states. Moreover, many chemical reactions proceed through unstable charged or radical intermediates, which also often have a multireference character.

The recently reported QED-CASCI approach^26^ and QED-2-RDM methods are well suited for describing strong correlation, but both have important limitations. QED-2-RDM methods have polynomial scaling with respect to the active space size, but so far, these variational approaches are designed to simulate the lowest energy states of a given spin symmetry, which are of limited utility for studying polariton states.^32^ The QED-CASCI method can be used to compute multiple states, but scales exponentially with the active space size, putting a hard limit of less than 20 correlated orbitals.^26^ The exponential scaling of CASCI is usually surpassed by using approximations to such as Selected-CI,^34−36^ heat-bath CI,^37^ full-CI QMC,^38^ or DMRG.^39−41^ To this end, we present a QED extension to the Density Matrix Renormalization Group method (QED-DMRG) that can provide efficient controlled approximations to numerically exact solutions to the Pauli–Fierz Hamiltonian.^15^ QED-DMRG also provides a powerful representation for approximating multiconfigurational multicomponent wave functions with similar accuracy and significantly lower computational cost than the recently reported QED-CASCI approach.

In what follows, we briefly review the basics of the DMRG method and outline the extensions necessary for QED-DMRG. Since our DMRG implementation (MOLMPS program^42^) employs the renormalized operators rather than the matrix product operators (MPOs), the presentation follows the original renormalization group picture.^43^ In order to demonstrate the accuracy of QED-DMRG, we present its application on the eigenstates of the Pauli–Fierz (PF) Hamiltonian tuned to the excitation from the ground to the first excited singlet state (S_0_ to S_1_) of *n*-oligoacenes with *n* ∈ ⟨2, 5⟩. For a discussion on the excited states of *n*-oligoacenes using a broad spectrum of excited-state theoretical approaches, we refer the reader to ref (44). and references therein.

## 2 Theory

### 2.1 Quantum Chemical DMRG Method

While the Density Matrix Renormalization Group (DMRG) method has origins in solid state physics,^43,45^ it is already established also in the realm of quantum chemistry,^39−41^ sometimes abbreviated as QC-DMRG. Here the DMRG algorithm is used to find the eigenstates of the electronic Hamiltonian
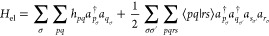
1where *h*_*pq*_ and ⟨*pq*|*rs*⟩ denote standard one and two-electron integrals in the molecular orbital (MO) basis, and σ and σ′ denote spin, σ, σ′ ∈ {↑, ↓}. Throughout this work, we assume the molecular orbitals are the same for both spins, and as a result the one and two-electron integrals are spin independent.

The DMRG method is based on the Matrix Product State (MPS)^46^ wave function ansatz, in which the exact FCI wave function (in the occupation basis representation)
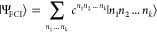
2is factorized into a linear tensor network
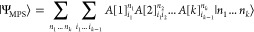
3The tensors *A*[*j*] have three indices. One physical, here labeled *n*_*j*_, corresponding to a physical degree of freedom. In quantum chemistry these are the possible occupations of a single molecular orbital (| ⟩, |↑⟩,|↓⟩,|↑↓⟩), leading to a dimension of four. The other two (*i*_*j*–1_, *i*_*j*_) are called virtual, and they are contracted with the neighboring tensors. The dimension of these indices, called the bond dimension, is the main parameter controlling both the accuracy and the computational cost.^40^ Since the bond dimension is in practical approximate calculations bounded, the number of DMRG variational parameters is, in contrast to FCI, polynomial.

The optimization of the individual tensors in the network is then performed sequentially in a process called sweeping. The standard two-site algorithm is based on the separation of the tensor network into four parts, the two sites taken explicitly (*j*, *j* + 1), all the sites to the left of the explicit sites (*i* < *j*, so-called left block) and the sites on the right (*i* > *j* + 1, right block). It provides the wave function in the two-site MPS form^46^
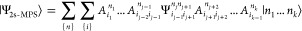
4where we have for simplicity omitted the MPS tensor square brackets with the site indices. The uncontracted virtual indices on the left and right blocks form the orthonormal many-particle bases ({|*l*⟩}, {|*r*⟩}), which span a subspace of the full Hilbert space of a given block. The orthonormality stems from the fact that the MPS tensors formed during the DMRG sweeping via the singular value decomposition (SVD, see below) fulfill the following conditions
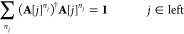
5
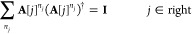
6

The four-index tensor Ψ_*i*_*j*–1_*i*_*j*+1__^*n*_*j*_*n*_*j*+1_^ in eq 4 is in each iteration of the sweeping procedure obtained by solving the Schrödinger equation projected onto the product space of the left block, two explicit sites, and the right block. The effective equation has, due to the orthonormality of the left and right block bases mentioned above, a form of a standard eigenvalue problem
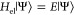
7which is solved by means of iterative solvers such as the Davidson algorithm.^47^ The wave function is expanded as
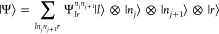
8Notice that |*l*⟩ labels basis states of the left block, which contains *j* – 1 sites, i.e. they correspond to the aforementioned uncontracted MPS virtual index *i*_*j*–1_.
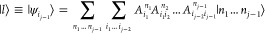
9Similarly |*r*⟩ corresponds to the uncontracted virtual index *i*_*j*+1_.

In DMRG, the explicit (determinant) representations of the complicated many-particle bases ({|*l*⟩}, {|*r*⟩}) are not stored, because it would lead to the original exponential scaling. Instead, the matrix representations of second-quantized operators needed for the action of the Hamiltonian on a wave function (see eq 7) are formed and stored. The matrix representations of a single site annihilation operators (in the {| ⟩, |↑ ⟩,| ↓⟩,|↑↓⟩} basis) read
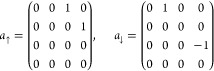
10When working with the enlarged left block (L) as a tensor product of the left block states with the states on the explicit left site and similarly the right enlarged block (R), the Hamiltonian for this bipartite splitting reads
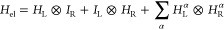
11where *H*_L/R_ represent the enlarged left/right block Hamiltonians, i.e. all indices in eq 1 belonging to the corresponding blocks and the last summation term represents the interaction between enlarged left and right blocks with indices split between both blocks. Let us demonstrate the main strategies on the example of the simpler one-electron Hamiltonian
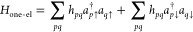
12Here, the interaction between the enlarged blocks consists of the two contributions
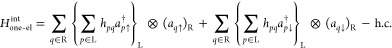
13In order to reduce the number of matrix–matrix multiplications during the action of the Hamiltonian on a wave function, which are the most CPU-demanding tasks, the efficient QC-DMRG codes work with the so-called pre-summed (or partially summed) operators,^48^ i.e. intermediates formed by contraction of operator matrices with MO integrals. In case of the one-electron Hamiltonian, eq 13, the enlarged left block one-electron pre-summed operators are encapsulated in the curly brackets and are defined as
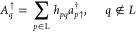
14
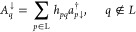
15The interaction terms are always composed of pre-summed operators on the one block and normal operators on the other block. In the case of one-electron Hamiltonian (13)
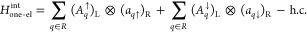
16Since the pre-summed operators are for efficiency reasons formed on the longer block, a switching between normal and pre-summed operators has to be done in the middle of each sweep.

The transition from one iteration of the sweep to another is carried out by means of the renormalization procedure. In this step, the basis of one of the blocks is, in the direction of the sweep, enlarged by one new site and the operators needed for the action of the Hamiltonian on the trial wave function are transformed into the new basis. The complementary block is, on the other hand, reduced by one site. This effectively moves us by one tensor in the MPS and the diagonalization of the effective Hamiltonian (eq 7) can be repeated. The exact basis obtained by enlarging by one site would be a tensor product between the original basis and the basis of the new site. This is unacceptable, as the size of the new basis would be a product of the sizes of the old basis and the basis of the added site. The choice of truncation of the new basis gave the name to the method, the basis is truncated so that the density matrix of the new block is changed as little as possible. This is done by diagonalizing the density matrix and keeping only the largest eigenvalues. When the wave function expansion coefficients Ψ_*lr*_^*n*_*j*_ *n*_*j*+1_^ (8) are reshaped into the matrix form ψ_(*l n*_*j*_), (*n*_*j*+1_ *r*)_, the aforementioned reduced density matrices can be computed in the following way
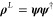
17
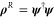
18For the transition to the next iteration, all operator matrices formed for the enlarged block have to be renormalized
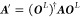
19where ***A*** represents an operator matrix in the nontruncated (4*M*-dimensional) basis and ***A***′ is the renormalized matrix representation in the truncated (*M*-dimensional) basis.

The procedure outlined above effectively performs an SVD decomposition on the wave function (8)
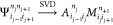
20where the new MPS tensor *A*[*j*] is formed and the three-leg tensor *M*[*j*+1] represents the rest of the SVD factorization. SVD in fact produces the best approximation of bipartite wave functions.^46^ The sum of the discarded eigenvalues is then called the Truncation Error (TRE).

### 2.2 Cavity QED-DMRG Method

The generalization of DMRG to multiple particle types is straightforward. The Hamiltonian now contains a mix of creation and annihilation operators corresponding to the different particle types, which separately satisfy own commutation/anticommutation rules. Each of the particle types is assigned its own set of sites in the MPS wave function and renormalized operators for all particle types, including the mixed terms describing the interaction between models, have to be formed. Previously, the QC-DMRG method has been generalized for the beyond-Born–Oppenhemier nuclear-electronic all-particle (NEAP) calculations^49,50^ and applied on the proton–electron problems, i.e. spin- Fermionic interactions. Herein, we generalize the QC-DMRG method for applications in polaritonic chemistry in which electronic (fermionic) and photonic (bosonic) degrees of freedom are treated on equal footing. Below, we give the details specific to this generalization.

We will restrict ourselves to the Pauli–Fierz (PF) Hamiltonian within the dipole approximation, which describes coupling of molecular systems to a single cavity (photonic) mode
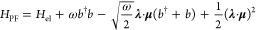
21where *H*_el_ is the electronic Hamiltonian (1), ω is the cavity photon frequency, **μ** represents the molecular dipole operator, **λ** is a coupling vector, and *b*^†^, *b* denote photonic creation and annihilation operators. The second term in eq 21 corresponds to the harmonic oscillator Hamiltonian of the bare cavity mode, the third term represents the so-called bilinear coupling, and the last one is the dipole self-energy term. We will assume the Cartesian coordinate system, thus **λ** and **μ** have *x*, *y*, and *z* components. It is convenient to define the scalar molecular dipole coupling operator as *d* = **μ**·**λ**.

Following the previous work on QED-CASCI,^26^ we compare two approaches differing by the input molecular orbitals. The simpler scenario corresponds to using the standard Hartree–Fock (HF) orbitals and searching for the eigenstates of the PF Hamiltonian presented in eq 21. In this case and similarly to eq 2, the QED-FCI wave function is expanded in the so-called particle number (PN) basis
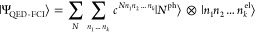
22where |*N*^ph^⟩ denotes the photonic PN basis, which comprises states |0⟩, |1⟩, |2⟩, ..., |*n*_max_⟩, *n*_max_ being the maximum photon occupation.

The second strategy is to perform the QED-HF calculation and transform from the PN basis into the coherent state (CS) basis^51,52^ by means of the unitary transformation
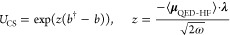
23where ⟨**μ**_QED-HF_⟩ is the expectation value of the dipole operator from a QED-HF calculation.

In order to keep the same wave function expansion as in eq 22, *U*_CS_ is applied at the level of the Hamiltonian (eq 21), which yields the PF Hamiltonian in the coherent state basis
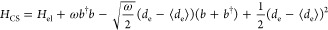
24where denoted as *d*_e_ and ⟨*d*_e_⟩ we keep only the electronic part of the dipole coupling operator *d* = **μ**·**λ** and of the coupling expectation value ⟨*d*⟩= ⟨**μ**_QED-HF_⟩·**λ**, because the nuclear parts cancel under the Born–Oppenheimer approximation.

We would like to point out that the reference state |0^ph^⟩⊗|ϕ_0_^el^⟩ transformed into the CS basis formally includes an infinite number of photon occupation states, due to the exponential form of *U*_CS_.^26^ Consequently, it was shown to outperform the PN basis in the correlated QED-CASCI calculations.^26^

### 2.3 Implementation Details

In adapting QC-DMRG for the PF Hamiltonian, the electronic part is identical to QC-DMRG, but we include a single photonic site. Herein, we have restricted ourselves to a single cavity mode, but the Hamiltonian (21), can be easily generalized for multiple cavity modes^25^ and generalization of the implementation described below is straightforward. Note that the dimension of the photonic site is not 4 as in the case of the electronic sites, but arbitrary, corresponding to the maximum number of photons included in the wave function.

In our cavity QED-DMRG implementation, we place the photonic site as the leftmost site in the chain. The advantage is two-fold. First, at any point over the sweep, the photonic operators are only in the left block. As a result, the rules for the combination of the operators on different blocks to form the Hamiltonian remain unchanged along the sweep. The second advantage is that the right block operators are identical to the QC-DMRG case and we can use the standard CI-DEAS warm-up procedure^53^ for the generation of electronic operators in the first sweep. We would like to note that a similar idea of using a single higher dimensional electronic site was used previously^54,55^ albeit in a different context.

The single-site matrix representation of the bosonic annihilation operator *b* in the basis {|0⟩, |1⟩, |2⟩, ...|*n*_max_⟩}, analogously to eq 10, has the matrix representation
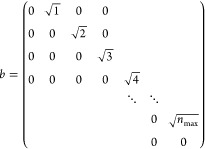
25and the creation operator *b*^†^is its Hermitian conjugate.

To derive the working form of the PF Hamiltonian, we group the terms containing creation and annihilation operators of only electrons, electrons and photons, and only photons. Here, we present this approach for the CS basis formulation (24). The corresponding PN basis formulation can be readily obtained with a few substitutions in the integrals.

The *H*_CS_ Hamiltonian can be rewritten as
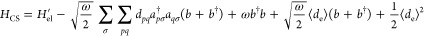
26where the modified electronic Hamiltonian has the same two-body structure as in eq 1, but with the following MO integrals
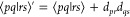
27
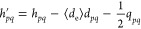
28and *d*_*pq*_ and *q*_*pq*_ represent modified electric dipole and electric quadrupole integrals given by
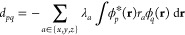
29
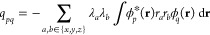
30As was mentioned above, only the left block carries the photonic operators, therefore the rules for building the action of the Hamiltonian on a trial wave function when employing the bipartite enlarged L–R splitting do not change along the DMRG sweep. The purely photonic operators (third and fourth terms in eq 26) can be added/absorbed into the left block Hamiltonian, which is coupled with an identity operator on the enlarged right block.

The second term in eq 26 needs to be separated into several contributions with a different number of operators acting on the right block
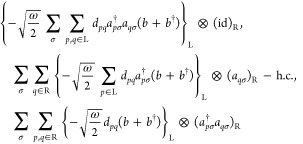
31where (id)_R_ is the identity operator on the right block.

In our pilot implementation, we do not switch between the normal and pre-summed operators in the middle of each sweep, but perform pre-summations only on the left block. This way, we can absorb the first contribution from eq 31 into the left block Hamiltonian. The left block part of the second contribution is then absorbed into *A*_*q*_^↑^ and *A*_*q*_^↓^ operators (14, 15), which are combined with a single annihilation operator on the enlarged right block. Similarly, the left block part of the third contribution can be absorbed into the pre-summed quadratic operators, which are coupled with quadratic operators on the enlarged right block.

Another thing to note is that DMRG in general works with the whole Fock space. This means that if we want to have a wave function with a given number of electrons, the number of electrons needs to be kept fixed throughout the calculation. This is done in the code by keeping track of the number of electrons and spin quantum numbers in each of the renormalized states, and allowing only the proper combinations into the wave function, both in QC-DMRG and QED-DMRG. On the other hand, no such thing is necessary for the photons, as generally the wave function will contain contributions from states with different numbers of photons, and also the renormalized states will mix the number of photons together. This is in stark contrast to works combining bosonic degrees of freedom with quantum chemical DMRG for non-Born–Oppenheimer calculations,^56^ which preserve the number of bosonic particles. In solid state physics, however, the Hubbard Holstein model is used, which does not preserve the number of phonons.^57−60^

## 3 Computational Details

To demonstrate the performance of the cavity QED-DMRG method, we performed all-π calculations of *n*-oligoacenes with *n* ranging from 2 to 5, i.e. naphthalene, anthracene, tetracene, and pentacene. The geometries used were optimized (similarly to ref (61)) at the UB3LYP/6-31G(d,p) level. Both the geometries and the active space orbitals are given in the SI. For all subsequent calculations we used the cc-pVDZ basis set.^62^ Naphthalene, which was recently analyzed using the QED-CASCI method,^26^ served as a basis for establishing the convergence of QED-DMRG with QED-CASCI results. For higher acenes, we investigate how the results, particularly the energy gap between polaritonic states, scale with the number of aromatic rings.

The performance of QC-DMRG is well-known to depend heavily on the type of orbitals used and their ordering on the 1D lattice.^63^ To investigate the effect of orbital type on QED-DMRG, we compared the performance of two different sets of spatial orbitals. In addition to canonical orbitals, we employed Pipek–Mezey split-localized orbitals. The ordering of the orbitals was optimized using the Fiedler algorithm,^64−66^ based solely on the purely electronic part of the Hamiltonian. Due to our implementation, the photon site was fixed as the leftmost site in the chain.

Furthermore, we compared two different formulations of the PF-Hamiltonian presented in Section 2.2, namely the CS formulation (24) and the PN formulation (21). It is worth noting that in the case of QED-CASCI, the CS formulation was shown to perform better.^26^ The two-site DMRG calculations were initialized using the CI-DEAS warm-up procedure^53^ and employed either fixed bond dimensions or variable bond dimensions, achieving the predefined truncation error through the Dynamical Block State Selection (DBSS) approach.^67^

**Figure 1:**
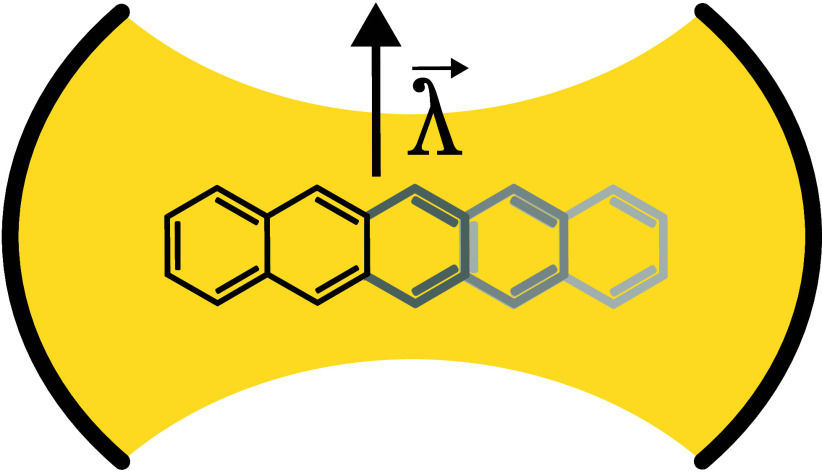
Orientation along the short axis of the oligoacene series, used throughout the majority of the calculations.

As mentioned above, the comparison with QED-CASCI has been done on the naphthalene molecule, with the photon energy tuned to S_0_-S_1_ transition energy of 0.160984 *E*_h_. We used a coupling vector oriented in plane along the short axis of the molecule, as shown in Figure 1. The size of the coupling varied from 0.005 to 0.2 atomic units (au).

Similarly for the larger acenes, where we only studied the behavior of the different states with the coupling strength, we used the photon energy equal to the S_0_-S_1_ transition at the zero coupling strength calculated with DMRG. This gave us an excitation energy of 0.14083 *E*_h_ for anthracene, 0.12741 *E*_h_ for tetracene and 0.11840 *E*_h_ for pentacene. We used a fixed bond dimension M = 1000. We compared two different orientations of the coupling vector, along the short axis of the molecule and along the long axis.

## 4 Results and Discussion

### 4.1 Verification against QED-CASCI

**Figure 2:**
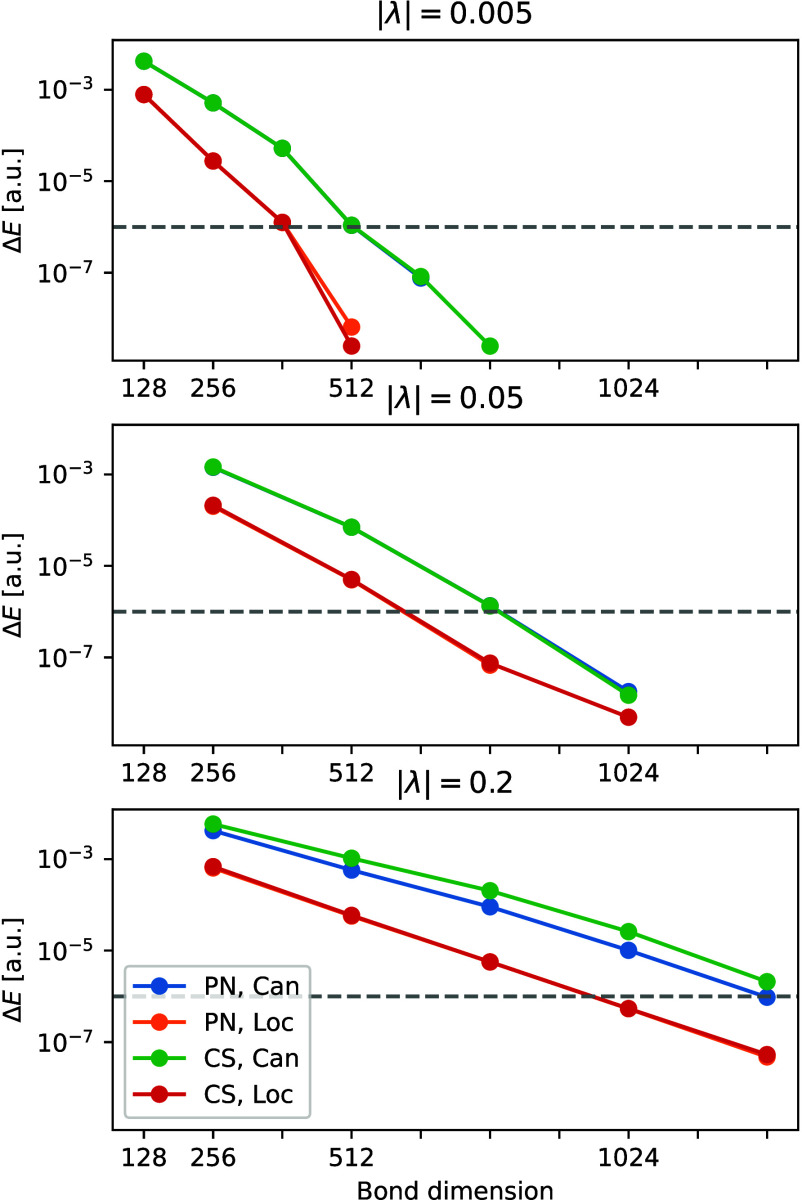
Comparison of the energetic errors in QED-DMRG with respect to exact QED-CASCI for the lower polaritonic state of naphthalene, using PN and CS Hamiltonian formulations in both canonical (Can) and split-localized MO (Loc) bases. Results are shown for varying bond dimensions and three coupling strengths. The orange and red curves overlap in all three plots. Similarly in the upper two panels, also the blue and green curves fully overlap.

Let us begin by comparing QED-DMRG and QED-CASCI on the naphthalene molecule. Figure 2 illustrates the energetic error of QED-DMRG relative to QED-CASCI for the lower polaritonic state across three different coupling strengths. Increasing the bond dimension allows us to reduce the error below our convergence threshold of 10^–6^*E*_h_ for DMRG. The almost linear dependence in a semilogarithmic plot hints at a roughly exponential decrease of the error with an increasing bond dimension.^39,68,69^ One can observe that with increasing coupling strength, the error for a given bond dimension grows due to greater entanglement between photonic and electronic degrees of freedom.

In Table 1 we show the truncation error and the RMSD of the five lowest states with respect to the reference CASCI depending on the magnitude of the cavity coupling. Similarly as with the error in energy, the truncation error grows with increasing the coupling strength. However, the growth is not very dramatic, with less than a 20% increase of the TRE with an order of magnitude stronger coupling.

**Table 1:** Truncation Error (TRE) of Naphthalene with a Bond Dimension of 256 for Different Values of the Coupling Strength and the Root Mean Square Deviation (RMSD) of Energies of the 5 Lowest States from the Exact CASCI Energy^a^

lambda (au)	0	0.005	0.01	0.02	0.03	0.04	0.05
Can-TRE	2.12·10^–3^	2.12·10^–3^	2.17·10^–3^	2.21·10^–3^	2.26·10^–3^	2.35·10^–3^	2.45·10^–3^
Loc-TRE	7.28·10^–5^	1.49·10^–4^	1.51·10^–4^	1.52·10^–4^	1.60·10^–4^	1.68·10^–4^	1.77·10^–4^
Can-RMSD	5.71·10^–4^	5.64·10^–4^	5.63·10^–4^	6.34·10^–4^	6.87·10^–4^	7.51·10^–4^	8.21·10^–4^
Loc-RMSD	2.60·10^–8^	7.12·10^–5^	7.37·10^–5^	8.02·10^–5^	9.10·10^–5^	9.65·10^–5^	1.04·10^–4^

a This has been done with the PN formulation and two different sets of spatial orbital bases, canonical (Can) and Pipek–Mezey split localized (Loc).

### 4.2 Factors Affecting the QED-DMRG Method

We have also studied other properties of the QED-DMRG method. Similarly to QC-DMRG, we can see both in Figure 2 and Table 1 that the truncation error and consequently the energy error are strongly reduced by localization of the active orbitals, the truncation error being an order of magnitude lower with the same bond dimension. Thus, for calculations of higher acenes, we have employed the split-localized orbitals. Overall, we found that for naphthalene with a split-localized basis we can achieve sub-millihartree accuracy with respect to QED-CASCI quite easily, even with a rather small bond dimension of 256.

The physical explanation for the superior performance of the localized orbitals is the local nature of electron correlation. In the local basis, the spatially separated orbitals are not correlated. This, together with a proper ordering of the sites in the MPS chain, leads to a much smaller tensor size needed for a given accuracy. From the plot it might seem that there is a difference in the performance of the PN formulation and the CS formulation. This however is caused simply by the suboptimal performance of DMRG in the canonical orbital basis. The slight difference between the canonical RHF and QED-RHF orbitals leads to a difference in accuracy between the PN and CS formulations. However, the origin is in the different orbital basis and not in the formulations themselves and localizing the orbitals makes the two formulations equivalent in terms of accuracy.

**Figure 3:**
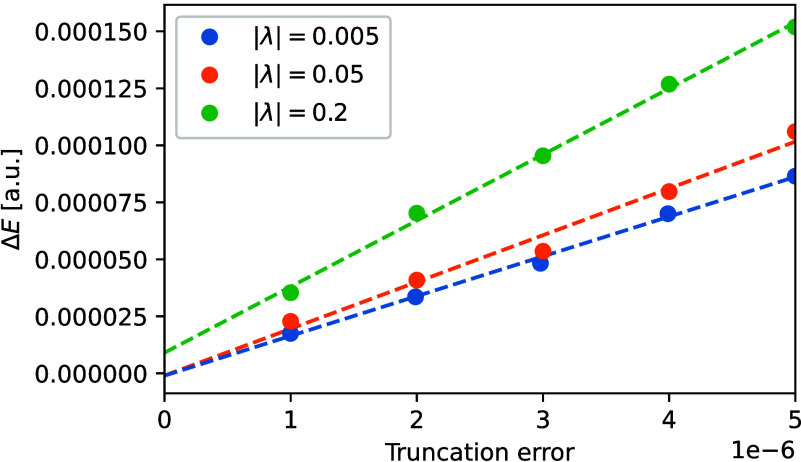
Dependence of the error in energy with the truncation error for the polaritonic state of naphthalene with different coupling strengths. This dependence is fitted with an affine function for the extrapolation to a zero truncation error.

Figure 3 shows the dependence of the energy error on the TRE set for various coupling strengths. A linear trend is observed for small TRE values, similar to QC-DMRG, which enables extrapolation of the energy to a zero TRE limit, which should in theory be equivalent to the exact CASCI energy. This extrapolation is done by plotting the total energy against the truncation and fitting with an affine function of the form *f*(*x*) = *ax* + *b*, where the coefficient *b* becomes our extrapolated energy. In our case in Figure 3, since we know the exact CASCI energy, we plot the error with respect to CASCI instead, and the resulting *b* is the error of the extrapolated energy instead. The errors of the extrapolated energies are −1·10^–6^, –1·10^–6^, and 9·10^–6^ atomic units for coupling values of |λ| = 0.005 au, |λ| = 0.05 au, and |λ| = 0.2 au, respectively. These errors are significantly lower than those of the most accurate calculations used for the extrapolation, which had a TRE of 1 ·10^–6^ (leftmost set of points in Figure 3). The errors of these calculations for the different coupling strengths were 2·10^–5^, 2·10^–5^, and 5·10^–5^*E*_h_ respectively.

**Figure 4:**
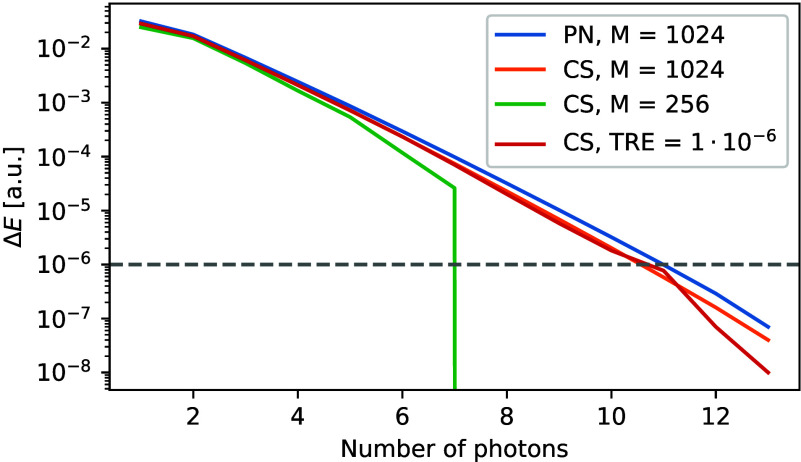
Energy difference between the polaritonic state with a given number of photons and the converged state with 14 photons, illustrating the saturation of the naphthalene wave function with photons. PN denotes the particle number basis, and CS denotes the coherent state basis.

In Figure 4 we show the error in energy of the polaritonic state of naphthalene with respect to the maximum number of photons in the wave function. The error is calculated for the coupling strength λ = 0.2 au as a difference between the energy with a given number of photons and the energy with 14 photons, which we considered converged. We can observe that the error decreases roughly exponentially with the number of photons, similar to ref (26). Nevertheless, the approximate nature of DMRG introduces additional features worth discussing. Using a bond dimension of 1024, which yields energies almost identical to the reference CASCI, we observe proper scaling across the entire range of values. Conversely, reducing the bond dimension to 256 limits the flexibility of the wave function, making it unable to fully account for the number of photons. As such, the system artificially gets saturated with a much smaller number of photons. Increasing the number of photons beyond this saturation limit results in nearly no changes to the resulting wave function and the change in energy abruptly drops to zero. Similarly, with DBSS the resulting curve follows the scaling until the energy error from the photon truncation does not reach roughly 10^–6^, which would be roughly the expected accuracy of a calculation with a fixed truncation error of 10^–6^. The convergence does not differ very significantly between the photon number and coherent basis formulations, however, the coherent formulation has the advantage of being translationally invariant, which in the photon number basis does not hold for charged molecules, unless we include enough photons in the calculation to saturate the wave function.^18,26,52^ On the other hand, the coherent state formulation uses the QED-RHF orbitals, which change with the coupling strength, and requires selecting an active space for every coupling vector separately, unlike with canonical RHF orbitals.

**Figure 5:**
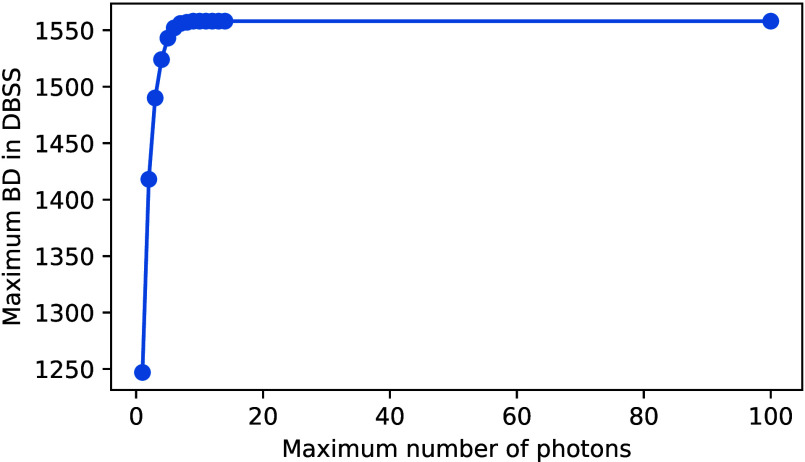
Maximum bond dimension required for a given maximum number of photons, illustrating the renormalization of unnecessary degrees of freedom. The analysis is based on naphthalene with a coupling strength of 0.2 au and a target truncation error (TRE) of 10^–6^.

One advantage of the DMRG method we would like to stress is that the extra photons in the basis, which do not contribute to the wave function, are removed in the renormalization procedure, and thus mean a very small extra computational cost. This is in sharp contrast with QED-CASCI, where every extra photon inevitably means an increase in the Hamiltonian matrix size. To demonstrate this, in Figure 5 we show the maximal bond dimension for DBSS for each number of photons. There we can clearly see that increasing the number of photons after saturating the wave function has no effect on the total number of renormalized basis states, even when going to the extreme limit of allowing up to 100 photons.

### 4.3 Results on the Oligoacene Series of Molecules

**Figure 6:**
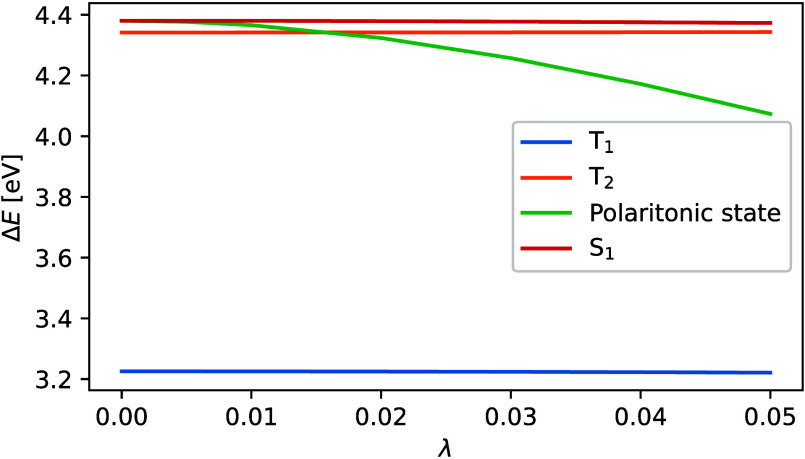
Energy differences between the ground state and excited states of the naphthalene molecule at a given coupling strength.

To explicitly show the polaritonic splitting of the S_1_ excited state of naphthalene, in Figure 6 we plot the dependence of excitation energies on the coupling strength. We find that, except for the split polaritonic state, the other states are affected by the interaction with the field equally, and thus the energy difference stays constant over the range. The split polaritonic state is then lowered in energy with respect to the other states, which can lead to states crossing, as can be seen for the S_1_ and T_2_ states in this case. The primary determinantal contributions to the resulting polaritonic state of naphthalene for λ = 0.05 au, with absolute CI coefficients greater than 0.01, are
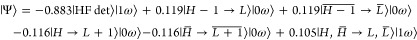
32where *H* and *L* denote the highest occupied molecular orbital (HOMO) and the lowest unoccupied molecular orbital (LUMO).

**Figure 7:**
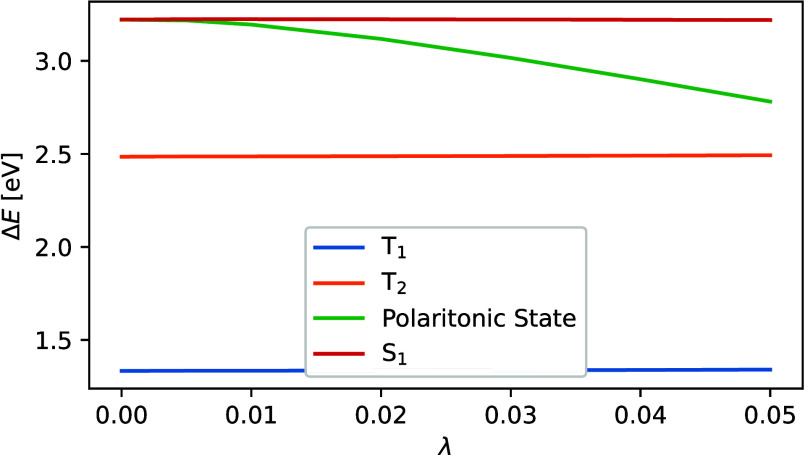
Energy differences between the ground state and excited states of the pentacene molecule at a given coupling strength.

Similarly, we show the value of the polaritonic splitting of pentacene in Figure 7. Although the polaritonic splitting increases with the number of aromatic rings, due to the larger S_1_ – T_2_ gap, the state inversion occurs at higher coupling strengths.

**Figure 8:**
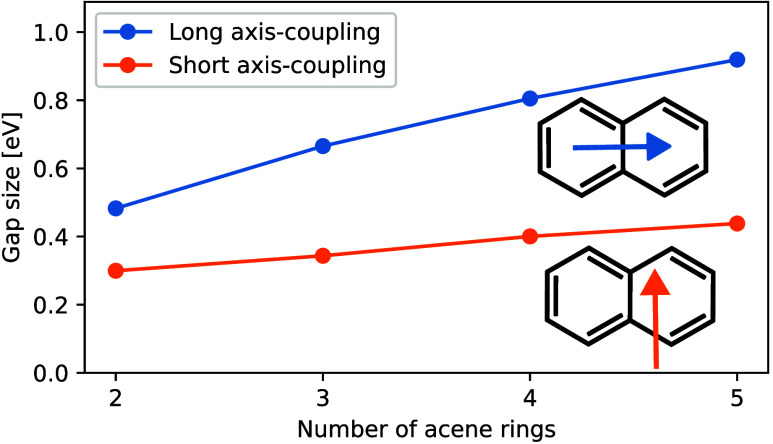
Energetic splitting of the polaritonic states for different numbers of acene rings at a fixed coupling strength of |λ| = 0.05 au. We compare two different possible orientations of the coupling vector.

By plotting the splitting value for a fixed coupling strength of |λ| = 0.05 au in Figure 8, we observe a remarkable linear increase with the number of rings, despite the underlying complexity of the processes involved. This trend, which is more pronounced for the coupling oriented in plane along the long axis, highlights the influence of molecular structure on polaritonic behavior and suggests potential avenues for tuning the properties of polycyclic aromatic hydrocarbons through molecular design. Since the density of excited states increases with an increasing number of acene rings and the splitting is caused by the cavity states coupling to excited states of the molecule, a plausible explanation of the increasing gap for the larger acenes would be that more excited states become energetically available for coupling to the cavity and lowering the energy of the polaritonic state. For reference, we also performed similar calculations for a strong coupling |λ| = 0.2 au for one of the orientations with similar results. These results are in the SI.

Unfortunately, we are not aware of any experimental studies on oligoacenes coupled to an optical resonator. However, simply comparing the excitation energies in our study obtained from QC-DMRG to experimental valued, which is done in Table 2, shows that DMRG is able to predict the general trend of a lowering S_0_-S_1_ transition energy with an increasing number of rings. Nevertheless, as has been already shown earlier,^61,77^ to achieve exact agreement with experimental values, it is crucial to account for dynamical correlation. Popular choices with DMRG then include Adiabatic Connection (AC),^61^ Pair Density Functional Theory,^77^ Tailored Coupled Clusters (TCC),^78^ or second-order *n*-electron valence state perturbation theory (NEVPT2).^79,80^ However, we would like to note that this is beyond the scope of this paper and will be a subject of future studies.

**Table 2:** Comparison of the S_0_–S_1_ Transition Energies (eV) from QC-DMRG in Our Study to Experimental Values

	S_0_–S_1_ (DMRG)	expt
naphthalene	4.38	3.97,^70^ 4.0^71^
anthracene	3.83	3.84,^72^ 3.45^73^
tetracene	3.47	2.6(0.11),^74^ 2.72^75^
pentacene	3.22	2.3^76^

## 5 Conclusions

In conclusion, we have developed a novel method for cavity-QED calculations utilizing the DMRG algorithm, which facilitates near exact CASCI calculations with significantly larger active spaces than traditional canonical-CASCI method. This approach is particularly suitable for systems with strong electronic correlation, especially in the regime of strong coupling to the photonic degrees of freedom. We have demonstrated the method’s capabilities on the *n*-oligoacenes series, with *n* ranging from 2 to 5, successfully managing up to 22 fully correlated π orbitals in pentacene. Our numerical results indicate that employing a split-localized coherent state basis yields fastest convergence toward the exact results. Additionally, we have shown that in the acenes series, the polaritonic splitting increases almost linearly with the number of aromatic rings, highlighting the influence of molecular structure on polaritonic behavior.

## Supporting Information Available

Oligoacene geometries used in the calculations, active spaces for the oligoacene molecules, and opening of the polaritonic state gap for the 0.2 coupling (PDF)
