# Supplementary material for: Polaritonic Chemistry Using the Density Matrix Renormalization Group Method
Source: J Chem Theory Comput. 2024 Oct 23;20(21):9424–34. doi: 10.1021/acs.jctc.4c00986 (PMC11562376; doi:10.1021/acs.jctc.4c00986)
Supplement: Supplementary file 1 — ct4c00986_si_001.pdf [file ct4c00986_si_001.pdf]

Supporting Information: Polaritonic Chemistry using the Density Matrix Renormalization Group Method

Mikuláš Matoušek,[*,†,‡] Nam Vu,[¶] Niranjan Govind,[§,‖] Jonathan J. Foley IV,[*,¶] and Libor Veis[*,†]

†*J. Heyrovský Institute of Physical Chemistry, Academy of Sciences of the Czech Republic, v.v.i., Dolejškova 3, 18223 Prague 8, Czech Republic*

‡*Faculty of Mathematics and Physics, Charles University, 12116 Prague 2, Czech Republic*

¶*Department of Chemistry, University of North Carolina Charlotte, Charlotte, North Carolina 28223, United States*

§*Physical and Computational Sciences Directorate, Pacific Northwest National Laboratory, Richland, Washington 99352, United States*

‖*Department of Chemistry, University of Washington, Seattle, Washington 98195, United States*

E-mail: mikulda@volny.cz; jfoley19@charlotte.edu; libor.veis@jh-inst.cas.cz

Oligoacene geometries used in the calculations.

Naphtalene

6	1.244884	1.402273	0.000002
1	-1.242596	2.489371	-0.000005
1	-3.377248	-1.245758	0.000002
1	-1.242596	-2.489371	0.000005
6	-2.433451	-0.708287	0.000001
6	-2.433451	0.708287	-0.000002
6	0.000000	0.717205	0.000000
1	3.377248	1.245759	0.000002
1	-3.377248	1.245758	-0.000004
6	0.000000	-0.717205	0.000000
6	-1.244884	-1.402273	0.000002
1	3.377248	-1.245759	-0.000003
6	-1.244884	1.402273	-0.000001
6	1.244883	-1.402273	-0.000002
1	1.242596	-2.489371	-0.000005
1	1.242597	2.489371	0.000005
6	2.433451	0.708287	0.000001
6	2.433451	-0.708287	-0.000002

Anthracene

6	-2.479178	1.406931	0.000000
6	-1.223620	0.722659	-0.000000
6	-0.000000	-1.403686	-0.000000
6	-1.223619	-0.722658	-0.000000
1	0.000000	2.491459	-0.000000
1	-2.476807	-2.493850	-0.000000
6	-2.479176	-1.406931	-0.000000
1	-4.606037	1.246319	0.000001
6	-3.659998	0.713027	0.000000
6	3.659997	-0.713028	0.000001
1	-2.476808	2.493850	0.000001
6	-3.659997	-0.713028	0.000000
1	-4.606036	-1.246321	-0.000000
6	0.000000	1.403688	-0.000000
6	1.223621	0.722659	-0.000000
6	1.223620	-0.722658	-0.000000
1	4.606037	1.246319	0.000000
1	-0.000000	-2.491457	-0.000000
6	2.479178	1.406930	-0.000000
1	2.476808	2.493849	-0.000000
6	2.479176	-1.406931	0.000000
6	3.659998	0.713027	0.000000
1	2.476807	-2.493850	0.000000
1	4.606036	-1.246321	0.000001

Tetracene

6	-3.710699	-1.409164	0.000008
6	-2.450123	-0.725956	0.000004
6	-1.235214	1.406510	-0.000003
6	-2.450124	0.725957	-0.000002
1	-1.235190	-2.494147	0.000017
1	-3.708733	2.496049	-0.000013
6	-3.710700	1.409163	-0.000009
1	-5.835073	-1.246965	0.000005
6	-4.888172	-0.715272	0.000003
6	2.450124	0.725957	0.000004
1	-3.708732	-2.496050	0.000019
6	-4.888173	0.715271	-0.000009
1	-5.835074	1.246963	-0.000016
6	-1.235214	-1.406509	0.000004
6	0.000000	-0.726284	0.000003
6	-0.000000	0.726285	0.000003
6	4.888173	0.715271	0.000002
1	-1.235191	2.494148	0.000002
6	1.235214	-1.406509	-0.000003
1	1.235190	-2.494147	0.000002
6	1.235214	1.406510	0.000004
6	2.450123	-0.725956	-0.000002
1	1.235191	2.494148	0.000018
6	3.710699	-1.409164	-0.000009
1	5.835073	-1.246965	-0.000016
6	4.888172	-0.715272	-0.000009
1	3.708732	-2.496050	-0.000013
6	3.710700	1.409163	0.000008
1	5.835074	1.246963	0.000004
1	3.708733	2.496049	0.000020

Pentacene

6	2.467268	-1.408059	0.000004
6	1.226311	-0.728680	0.000015
6	-0.000001	1.408625	0.000011
6	1.226311	0.728681	0.000015
1	0.000002	-2.496122	0.000070
1	2.467517	2.495667	0.000048
6	2.467268	1.408061	0.000004
1	4.939350	-2.497204	0.000002
6	3.678063	-0.727692	-0.000001
6	-3.678062	0.727693	0.000001
1	2.467518	-2.495666	0.000049
6	3.678063	0.727694	0.000000
1	7.064249	1.247294	-0.000043
6	-0.000000	-1.408626	0.000012
6	-1.226310	-0.728682	0.000018
6	-1.226310	0.728680	0.000017
6	-6.116910	0.716423	-0.000027
1	-0.000000	2.496120	0.000067
6	-2.467269	-1.408061	0.000008
1	-2.467518	-2.495667	0.000051
6	-2.467269	1.408059	0.000007
6	-3.678063	-0.727694	0.000002
1	-2.467518	2.495666	0.000049
6	-4.941087	-1.410338	-0.000010
1	-7.064251	-1.247292	-0.000041
6	-6.116911	-0.716421	-0.000027
1	-4.939352	-2.497205	0.000004
6	-4.941086	1.410338	-0.000011
1	-7.064250	1.247295	-0.000042
1	-4.939350	2.497205	0.000003
6	4.941086	-1.410337	-0.000010
6	4.941086	1.410337	-0.000007
1	4.939351	2.497205	0.000006
6	6.116911	-0.716422	-0.000026
6	6.116911	0.716421	-0.000025
1	7.064249	-1.247296	-0.000046

Active spaces for the oligoacene molecules

(After split localization, isovalue 0.01)

Naphtalene

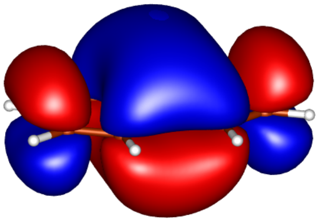
1.

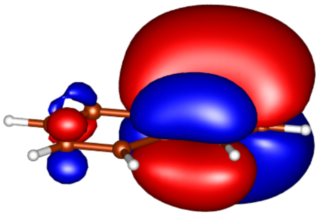
2.

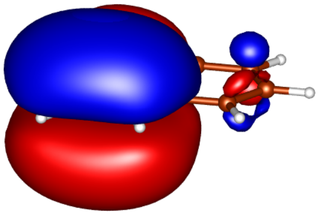
3.

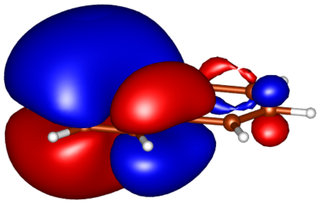
4.

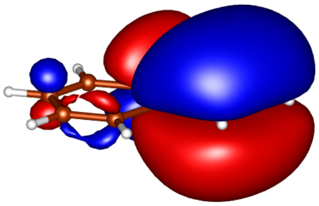
5.

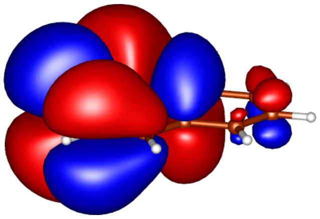
6.

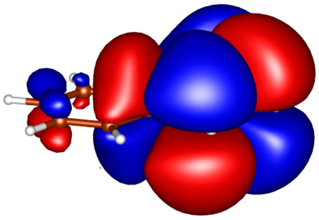
7.

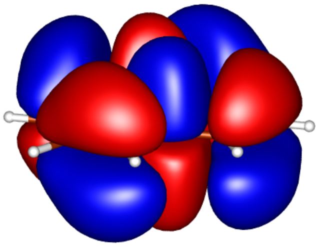
8.

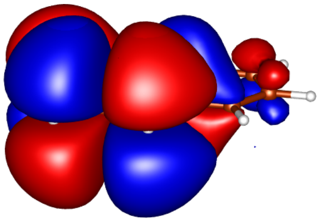
9.

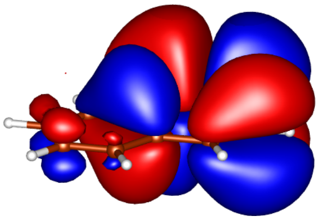
10.

Anthracene

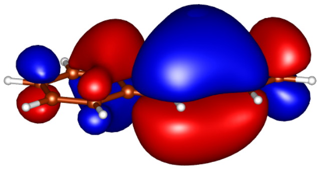
1.

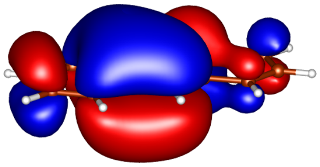
2.

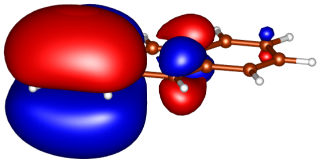
3.

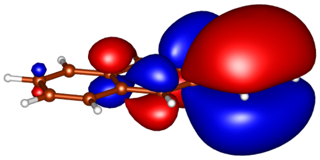
4.

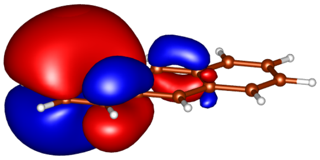
5.

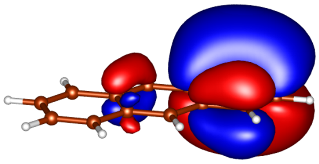
6.

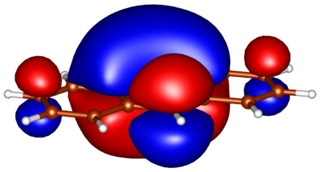
7.

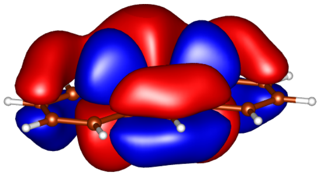
8.

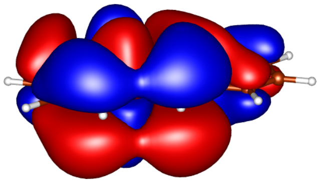
9.

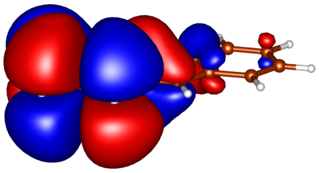
10.

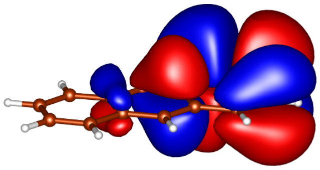
11.

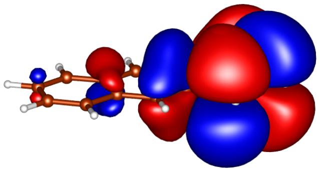
12.

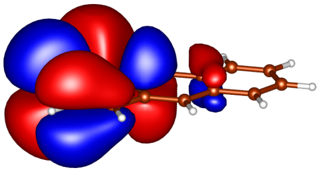
13.
-

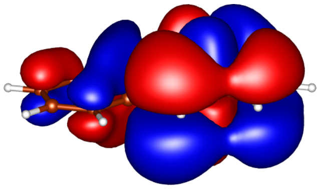
14.

Tetracene

1. 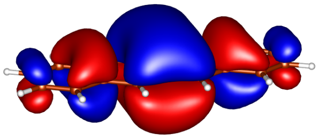

2. 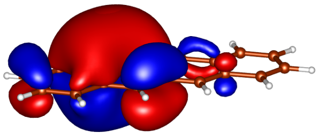

3. 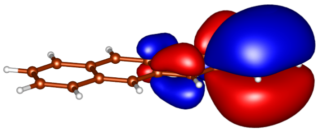

4. 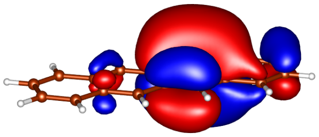

5. 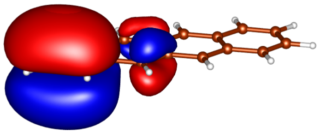

6. 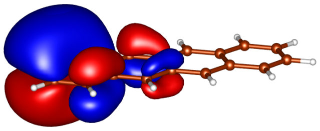

7. 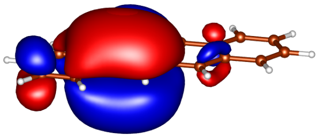

8. 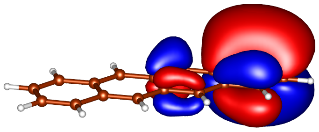

9. 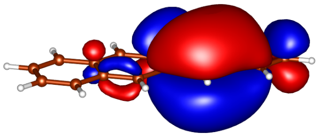

10. 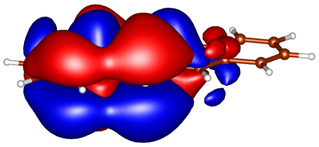

11. 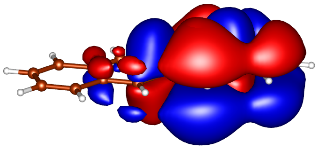

12. 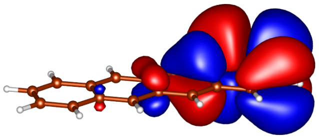

13. 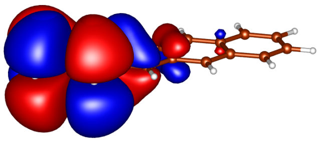

14. 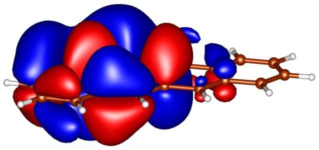

15. 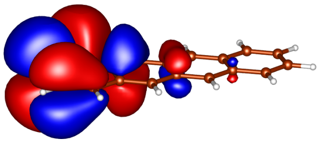

16. 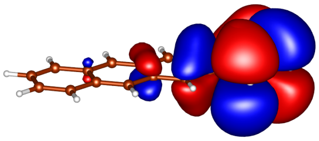

17. 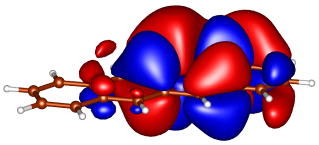

-

18. 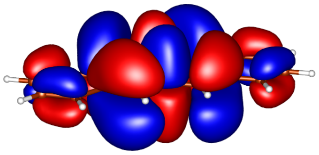

Pentacene

1.
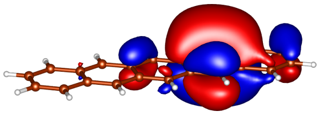
2.
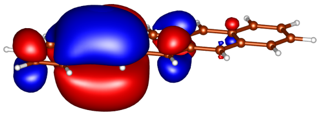
3.
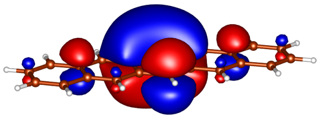
4.
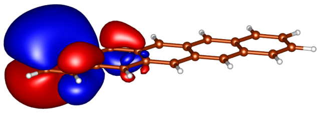
5.
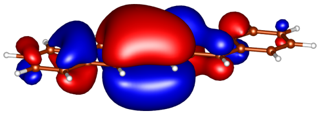
6.
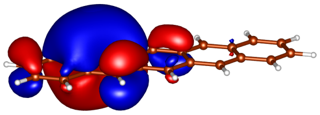
7.
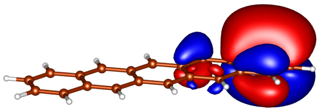
8.
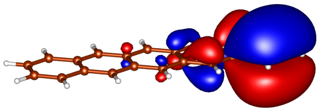
9.
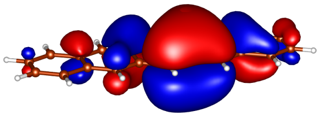
10.
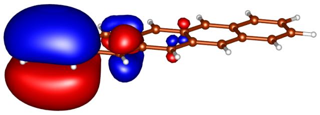
11.
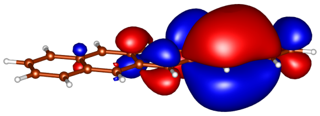
12.
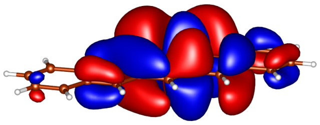
13.
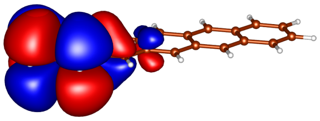
14.
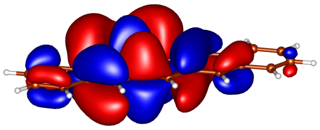
15.
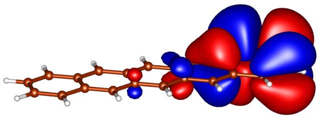
16.
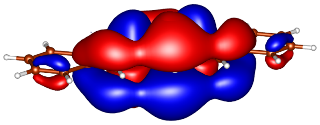
17.
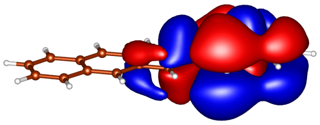
18.
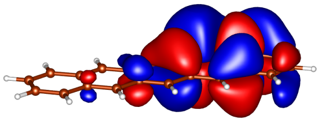
19.
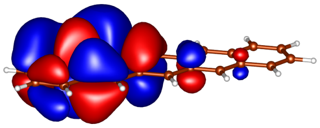
20.
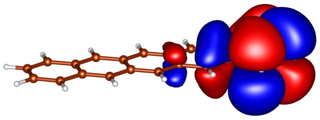
21.
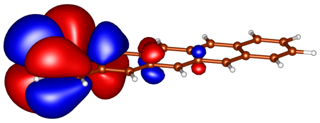
22.
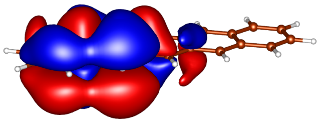

Opening of the polaritonic state gap for the 0.2 coupling

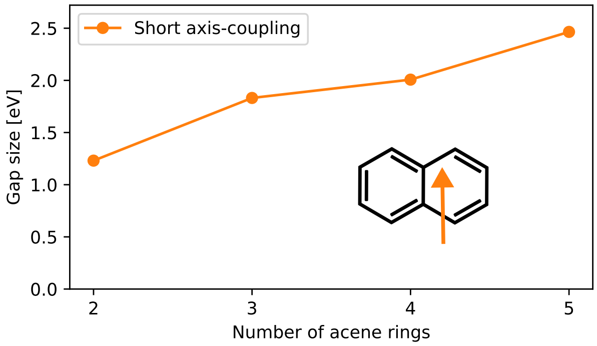

Figure S1: Energetic splitting of the polaritonic states for different numbers of acene rings at a fixed coupling strength of $|\lambda| = 0.2$ a.u.. We show the orientation of the coupling vector along the long axis of the molecule.
